# Adjunctive Cerebrolysin therapy following mechanical thrombectomy for acute basilar artery occlusion: a case report

**DOI:** 10.25122/jml-2026-0081

**Published:** 2026-06

**Authors:** Marina Roje Bedeković, Jerković Ivona, Marijana Bosnar Puretić

**Affiliations:** 1 University Department of Neurology, University Hospital Center Sestre Milosrdnice, Zagreb, Croatia; 2 School of Medicine, University of Zagreb, Zagreb, Croatia; 3 Faculty of Medicine, Catholic University of Croatia, Zagreb, Croatia

**Keywords:** adjunctive therapy, Cerebrolysin, cerebroprotection, posterior circulation stroke, basilar artery occlusion

## Abstract

Basilar artery occlusion (BAO) is an uncommon yet devastating cause of posterior circulation stroke, frequently resulting in severe disability or death despite successful reperfusion. We describe a 67-year-old woman with multiple vascular risk factors who presented with acute BAO and was treated with intravenous thrombolysis followed by successful mechanical thrombectomy (Thrombolysis in Cerebral Infarction [TICI] grade 3), with adjunctive Cerebrolysin therapy administered subsequently. Rapid neurological improvement was observed within 24 hours, with the National Institutes of Health Stroke Scale (NIHSS) score decreasing from 7 to 2, followed by complete neurological recovery (NIHSS 0) by day 3. Clinical recovery was sustained throughout the 12-month follow-up period, with only mild residual gait disturbance noted at discharge. No adverse events attributable to Cerebrolysin were observed. Successful reperfusion remains the key determinant of outcome in BAO, and no causal inference can be made regarding Cerebrolysin. This case adds to the limited evidence on the use of Cerebrolysin as an adjunct to reperfusion therapy and supports further investigation in controlled studies of posterior circulation stroke.

## Introduction

Basilar artery occlusion (BAO) is a rare but devastating subtype of posterior circulation stroke, associated with poor functional outcomes and reported mortality rates approaching 85% in untreated patients [1]. Recent randomized controlled trials (RCTs), including the ATTENTION and BAOCHE trials, have shown a benefit of mechanical thrombectomy in selected BAO patients, with intravenous thrombolysis (IVT) used as concomitant therapy [2,3]. Despite successful reperfusion, a substantial proportion of patients have unfavorable outcomes, highlighting an ongoing therapeutic challenge in BAO [2-5].

These limitations have prompted interest in adjunctive neuroprotective strategies to enhance neurological recovery. Cerebrolysin is a neuropeptide preparation with proposed multimodal cerebroprotective and neurotrophic properties and has been investigated as an adjunct to standard stroke therapy. Most clinical evidence comes from studies in anterior circulation stroke, where Cerebrolysin is well tolerated and may be associated with functional benefits [6-12]. However, data on posterior circulation stroke, including BAO, remain limited.

An ongoing prospective study evaluating Cerebrolysin as an add-on therapy for posterior circulation stroke secondary to BAO has been initiated at the Department of Neurology, University Hospital Center Sestre Milosrdnice (ClinicalTrials.gov: NCT06489925).

Here, we report a case from this study describing a favorable clinical outcome following intravenous thrombolysis (IVT), mechanical thrombectomy, and adjunctive Cerebrolysin therapy.

## Patient information

A 67-year-old woman with a history of hypertension, dyslipidemia, diabetes mellitus, and chronic right internal carotid artery occlusion following elective endarterectomy presented with acute-onset vertigo and left-sided hemiparesis.

There was no previous history of stroke or cognitive impairment. No relevant family history was reported.

## Clinical findings

On admission, the patient was somnolent but arousable, with preserved comprehension and mild dysarthria. Neurological examination revealed left internuclear ophthalmoplegia, vertical nystagmus, mild left-sided hemiparesis, and left-sided dysmetria. The patient was unable to stand or walk without assistance. The NIHSS score was 7. The clinical progression and treatment timeline are presented in Table 1.

**Table 1:** Clinical progression and treatment timeline

Day	Clinical Course and Key Therapeutic Interventions
Day 0 (~45 minutes before admission)	Sudden onset of left-sided limb weakness, dysmetria, somnolence, dysarthria, and internuclear ophthalmoplegia. NIHSS score 7.
Day 0 (upon admission)	IV alteplase at a standard dose of 0.9 mg/kg administered within one hour of hospital admission, followed by immediate endovascular mechanical thrombectomy. Cerebrolysin initiated 7 hours after symptom onset at a dose of 30 mL in 250 mL of 0.9% NaCl, administered IV once daily for 14 days.
Day 1	Mild paresis of the left upper limb and dysmetria in the left extremities observed on clinical assessment. NIHSS score 2. ASA 100 mg PO once daily, atorvastatin 80 mg PO once daily, and LMWH 40 mg SC once daily for DVT prophylaxis initiated alongside Cerebrolysin. Hyperglycaemia corrected as needed with short-acting subcutaneus insulin. Perindopril/ amlodipine 5mg/ 5mg PO once daily continued without dose adjustment.
Day 3	No neurological deficits detected. NIHSS score 0. Patient mobilized to upright position with physiotherapist assistance.
Day 5	Structured daily neurorehabilitation program (45-60 min) initiated, including coordination, mobility, balance, and activities of daily living (ADL)-focused training.
Day 16	Mild residual gait disturbance noted. mRS score 1. Patient discharged home on ASA 100 mg PO once daily, atorvastatin 80 mg PO once daily, adjusted antidiabetic therapy as per glycaemic control, and continuation of chronic antihypertensive therapy.

NIHSS, National Institutes of Health Stroke Scale; mRS, modified Rankin Scale; IV, intravenous; PO, per os; SC, subcutaneous; ASA, acetylsalicylic acid; LMWH, low-molecular-weight heparin; DVT, deep vein thrombosis

## Diagnostic assessment

**Figure 1:**
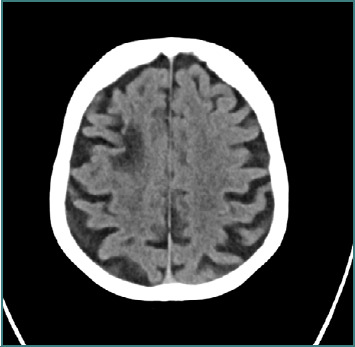
Non-contrast brain CT showed an encephalomalacia and gliotic scar in the right frontal lobe, consistent with prior ischemic injury

**Figure 2:**
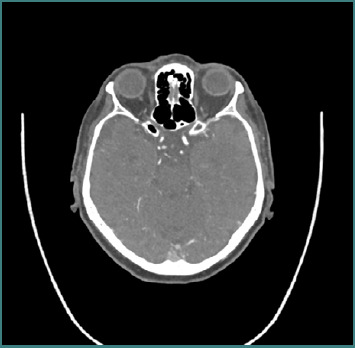
CT angiography demonstrated basilar artery occlusion (BAO)

Non-contrast brain CT showed no acute ischemia or intracranial hemorrhage, apart from an old encephalomalacic and gliotic scar in the right frontal lobe (Figure 1). CT angiography demonstrated basilar artery occlusion (Figure 2), with thrombus extending into both superior cerebellar arteries, and chronic occlusion of the right common and internal carotid arteries.

Laboratory evaluation revealed hyperglycemia (11.9 mmol/L); other parameters were within normal limits.

**Figure 3:**
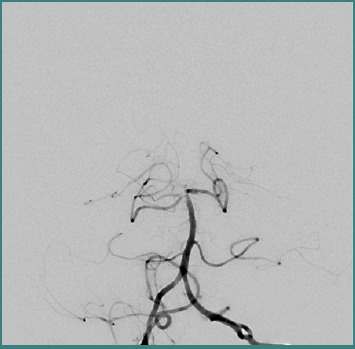
Digital subtraction angiography (DSA) showing partial recanalization of the basilar artery following thrombolytic therapy

**Figure 4:**
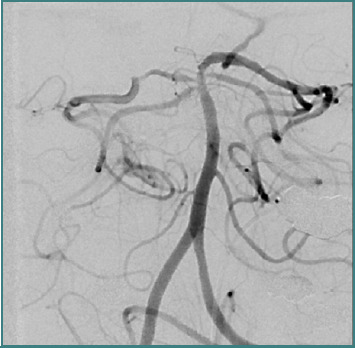
DSA showing subsequent complete recanalization (TICI grade 3) achieved by mechanical thrombectomy

Digital subtraction angiography (DSA) demonstrated partial recanalization of the basilar artery after thrombolysis (Figure 3), followed by complete reperfusion—Thrombolysis in Cerebral Infarction (TICI) grade 3 after mechanical thrombectomy (Figure 4).

**Figure 5:**
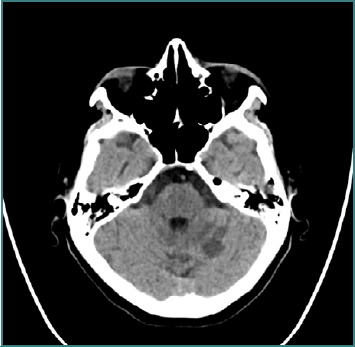
Non-contrast brain CT performed 24 hours after acute treatment, demonstrating acute ischemia in the left anterior cerebellar lobe and left anterior cerebellar peduncle, without evidence of intracranial hemorrhage

Follow-up brain CT showed acute ischemia in the left anterior cerebellar lobe and superior cerebellar peduncle without hemorrhagic transformation (Figure 5).

A comprehensive post-procedural diagnostic work-up was performed. The 24-hour Holter monitoring did not reveal atrial fibrillation or other clinically significant arrhythmias, and transthoracic echocardiography showed no significant structural abnormalities. The transcranial Doppler (TCD) bubble test was negative for right-to-left shunt, with no evidence of a patent foramen ovale or other intracardiac or extracardiac shunt as a potential embolic source. Carotid and vertebral artery Doppler ultrasonography demonstrated chronic occlusion of the right common carotid artery and right internal carotid artery, without evidence of acute or new large-artery pathology.

## Therapeutic intervention

Intravenous (IV) alteplase was administered within one hour of admission and was immediately followed by mechanical thrombectomy. Endovascular thrombectomy was performed via a right transradial approach. A guiding catheter was positioned in the proximal segment of the basilar artery. Angiography demonstrated partial recanalization of the basilar apex. Mechanical thrombectomy was then performed using a stent retriever in combination with an aspiration catheter, resulting in complete reperfusion (TICI 3). Hemostasis at the right radial puncture site was achieved by manual compression.

Therapy with Cerebrolysin (30 mL diluted in 250 mL saline, IV once daily for 14 days) was initiated 7 hours after symptom onset. The dosing regimen was based on commonly used protocols from previous acute ischemic stroke studies of Cerebrolysin, in which adjunctive Cerebrolysin therapy was typically initiated shortly after reperfusion therapy and administered at a dose of 30 mL IV once daily, with treatment durations ranging from 10 to 21 days in most trials [6-12].

Secondary prevention included acetylsalicylic acid, antihypertensive and lipid-lowering therapy, and prophylactic unfractionated heparin. A structured neurorehabilitation program was initiated on hospital day 5 and provided daily until discharge, with 45–60-minute sessions. The intervention included coordination training (Frenkel exercises and proprioceptive neuromuscular facilitation [PNF] rhythmic stabilization), task-specific training, functional mobility training [bed mobility, transfers, sit-to-stand activities, and fall prevention], and neuromuscular re-education focusing on balance, coordination, weight-bearing, and visual cueing strategies. Patient and caregiver education, as well as an individualized 24-hour rehabilitation program, were also implemented.

## Follow-up and outcomes

At 24 hours, the NIHSS score improved to 2, with mild left upper-limb paresis and dysmetria. By day 3, neurological examination was normal (NIHSS 0). At discharge (hospital day 16), a mild gait disturbance persisted (mRS 1).

No adverse effects related to Cerebrolysin were observed.

Follow-up at 1, 3, 6, and 12 months demonstrated sustained neurological stability. At the 12-month follow-up, the patient reported intermittent gait unsteadiness and peripheral vascular symptoms, which were managed conservatively.

## Discussion

This case illustrates rapid and sustained neurological recovery following successful mechanical thrombectomy for BAO in a patient who also received IVT and adjunctive Cerebrolysin therapy. The patient presented with an NIHSS score of 7 and achieved complete resolution of neurological deficits within 72 hours (NIHSS 0), with only mild residual gait impairment at discharge, resulting in a favorable functional outcome (mRS 1).

Endovascular thrombectomy has been shown to improve functional outcomes in BAO in both observational studies and RCTs. The BASILAR registry reported favorable functional outcomes following reperfusion, while recent RCTs demonstrated higher rates of good functional outcomes compared with medical management [2,3,13]. Both ATTENTION and BAOCHE demonstrated early neurological improvement after thrombectomy. ATTENTION reported lower median NIHSS scores at 24-72 hours (21 vs. 30) and at 5–7 days or discharge (16 vs. 35), while BAOCHE showed higher rates of dramatic early neurological improvement (25% vs 10%) [2,3]. Notably, these trials predominantly enrolled patients with higher baseline neurological severity (NIHSS ≥ 10), while data on outcomes in patients with lower NIHSS scores remain limited [2-4].

However, despite the overall benefits of reperfusion therapy, approximately half of the patients with BAO fail to achieve functional independence at 90 days despite successful reperfusion, reflecting the phenomenon of futile recanalization [2-4,13,14]. These variable outcomes have been associated with baseline neurological severity, time to reperfusion, complex vascular anatomy of the posterior circulation, and secondary ischemic injury processes [14-18].

In this context, Cerebrolysin was administered as an adjunctive therapy based on its proposed multimodal neurotrophic and neuroprotective properties, which are thought to reflect general pathophysiological pathways of ischemic brain injury rather than being specific to a particular cerebrovascular territory. Clinical evidence to date has predominantly been derived from anterior circulation stroke populations, and, in the absence of a known circulation-specific mechanism, its potential effects in posterior circulation stroke are hypothesized to be similar. However, this remains unproven [8,10,12,19,20].

Overall, the clinical course observed in this patient is consistent with the favorable end of the outcome spectrum reported after complete (TICI 3) reperfusion in BAO, where early and substantial neurological improvement is commonly driven by restoration of posterior circulation blood flow [2-4,13].

## CONCLUSION

Although adjunctive Cerebrolysin was administered as a cerebroprotective agent, its contribution cannot be disentangled from the effect of complete reperfusion, which remains the primary determinant of outcome.

Structured rehabilitation, including coordination, mobility, balance, and activities of daily living (ADL)-focused training, was initiated after early substantial recovery; its contribution to long-term functional stability cannot be excluded. The patient reported rapid symptom improvement and expressed satisfaction with the recovery process, particularly regarding regained mobility and independence.

This case highlights the need for further prospective studies evaluating the use of adjunctive Cerebrolysin alongside standard reperfusion therapies to clarify its potential cerebroprotective role in posterior circulation stroke.

## Data Availability

Further data is available from the corresponding author upon reasonable request.
